# An Improved Strategy for Detection and Localization of Nodules in Liver Tissues by a 16 MHz Needle Ultrasonic Probe Mounted on a Robotic Platform

**DOI:** 10.3390/s20041183

**Published:** 2020-02-21

**Authors:** Andrea Bulletti, Marina Mazzoni, Sahana Prasanna, Luca Massari, Arianna Menciassi, Calogero Maria Oddo, Lorenzo Capineri

**Affiliations:** 1 Department of Information Engineering, Università degli Studi di Firenze, 50139 Florence, Italy; andrea.bulletti@unifi.it (A.B.); m.mazzoni@ifac.cnr.it (M.M.); 2 Sant’Anna School of Advanced Studies, The BioRobotics Institute, 56025 Pisa, Italy; sahana.prasanna@santannapisa.it (S.P.); luca.massari@santannapisa.it (L.M.); arianna.menciassi@santannapisa.it (A.M.); calogero.oddo@santannapisa.it (C.M.O.); 3 Consiglio Nazionale delle Ricerche of Italy, Istituto di Fisica Applicata “Nello Carrara”, 50121 Florence, Italy

**Keywords:** ultrasonic probe, cancer nodules, detection, localization, agar phantom, liver tissue, ultrasound signal processing, correlation index

## Abstract

This study presents an improved strategy for the detection and localization of small size nodules (down to few mm) of agar in excised pork liver tissues via pulse-echo ultrasound measurements performed with a 16 MHz needle probe. This work contributes to the development of a new generation of medical instruments to support robotic surgery decision processes that need information about cancerous tissues in a short time (minutes). The developed ultrasonic probe is part of a scanning platform designed for the automation of surgery-associated histological analyses. It was coupled with a force sensor to control the indentation of tissue samples placed on a steel plate. For the detection of nodules, we took advantage of the property of nodules of altering not only the acoustical properties of tissues producing ultrasound attenuation, but also of developing patterns at their boundary that can modify the shape and the amplitude of the received echo signals from the steel plate supporting the tissues. Besides the Correlation Index Amplitude (CIA), which is linked to the overall amplitude changes of the ultrasonic signals, we introduced the Correlation Index Shape (CIS) linked to their shape changes. Furthermore, we applied AND-OR logical operators to these correlation indices. The results were found particularly helpful in the localization of the irregular masses of agar we inserted into some excised liver tissues, and in the individuation of the regions of major interest over which perform the vertical dissections of tissues in an automated analysis finalized to histopathology. We correctly identified up to 89% of inclusions, with an improvement of about 14% with respect to the result obtained (78%) from the analysis performed with the CIA parameter only.

## 1. Introduction

Ultrasound (US) systems are employed in medical diagnosis for the imaging of tumors [1,2,3,4,5], often used for early cancer detection [6,7]. Their practice is not expensive for the patient and is easy to use in a hospital ward and in medical clinics. Many of these applications avail a pulse-echo ultrasonic transducer array configuration [8] associated with B-mode techniques to visualize the organs of interest and their surroundings with brightness-scale imaging. During surgical interventions on cancer patients, US diagnostic techniques were proposed, associated with other instrumental techniques like Computed Tomography and Magnetic Resonance Imaging [9], Mammography [10,11] and Tactile Sensing [12]. Recently, we designed a prototype instrument for ex-vivo diagnosis availing of a pulse echoes overlap method [13,14,15,16] from a strong reflector. It was devoted to the mapping of an excised tissue during a surgery, which had to be intra-operatively analyzed by the pathologist before the end of the surgery, and further treated for a pathological analysis [17]. This was one goal of the IMEROS (Integrated MEdical-RObotic Solutions) project which focused on the study of new integrated robotic technologies for intraoperative tele-assisted diagnosis and for the surgical treatment of tumors. In this project, we proposed US techniques associated with tactile sensing to analyze stiffness and accurately localize tumor nodules [18]. Since the US methods for ex-vivo analyses had to operate with dry coupling in order to avoid contamination for the successive pathological analysis, we evaluated US imaging with commercial arrays that were not suitable. To mimic palpations of the resected malignant tissue (a general practice performed by a pathologist) we used a 16 MHz central frequency needle US probe with a load cell for the tactile analyses. Using a robotic platform, and machine learning techniques for classification, we detected and localized inclusions in phantoms mimicking the elastic and ultrasound properties of excised human tissues [19,20,21,22]. We carried out the experimental evaluation by means of agar-based phantoms suited to mimic soft tissues, either in their acoustic and mechanical properties. 

In this paper, we present a further step of the research toward a complete autonomous diagnostic method with experimentations performed on ex-vivo pork liver tissues, for their similarity to human ones, with dimensions comparable to those excised for biopsy. In fact, primary liver cancer (hepatocellular carcinoma or cholangiocarcinoma) is the sixth most common cancer worldwide and this organ can be treated using robotic surgery like the da Vinci robot, with a minimally invasive approach [23]. Furthermore, we used agar inclusions to mimic cancer lesions. Since the ultrasonic Correlation Index Amplitude (CIA) already tested on agar-based phantoms with spherical inclusions of higher agar density correctly identified only 56% of inclusions [18], we found this parameter alone insufficient to give a good statistical classification of agar nodules. Then we introduced a second correlation index, Correlation Index Shape (CIS), for the evaluation of the shape modifications of signals also. In fact, both CIA and CIS correlation indices are significant to alert for the presence of inclusions. Because amplitude and shape modifications did not necessarily occur in the same US probe position, we considered a study useful based on these two correlation indexes with the aim of improving the US statistical classification results. The strategy consisted of processing CIA and CIS combined in AND-OR logic. We show that this new approach provided complimentary maps of inclusions both in the agar phantoms and in the ex-vivo liver tissues. These maps can be used to define the contours of the inclusions with more reliability. 

At first for the validation of this new strategy, we used a previously fabricated agar phantom with spherical inclusions of different diameters [18] to mimic cancer nodules. The fixed position of inclusions inside the matrix and their designed dimensions allowed the evaluation of the CIA and CIS maps finalized to the individuation of nodules by the localization of the regions of maximum attenuation, and, possibly, in the determination of the nodule’s lateral extension. Then the application of the new strategy is applied to liver tissue samples with agar inclusions mimicking cancer nodules.

The paper is organized as follows: Section 2 describes the US sensor experimental set-up, the design and fabrication of phantoms and gives the experimental protocol to perform investigations on agar-phantoms and liver tissues. In Section 3, we report the US signal processing for the A-scan and the map generation of the investigated area. In Section 4, we show the results of ultrasound data analyses. In the final section, the discussion of the advantages introduced by the new strategy and future integrations of the US signal processing for diagnostics on ex-vivo tissues performed with a remotely accessible scanner. 

## 2. Experimental Procedure for US Inspection on Agar-phantoms and Liver Tissue

### 2.1. Experimental Set-Up

**Figure 1:**
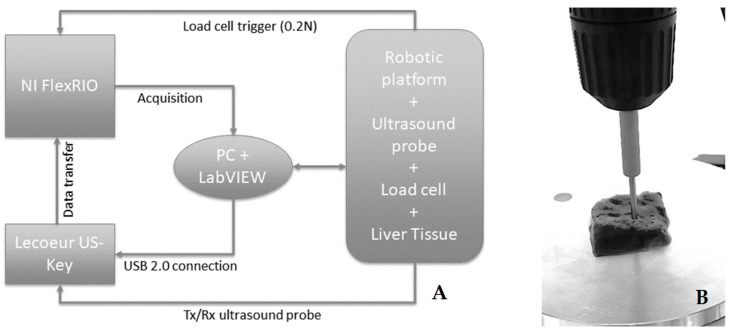
Block scheme of the experimental setup (**A**). Zoom on the ultrasonic needle probe (16 MHz central frequency, 3 mm external diameter) in contact with a liver sample integrated with the holder of the load cell for tactile sensing (**B**).

The probe is part of an automated system reported in [18] and shown in Figure 1.

The ultrasound probe (Sonomed, mod. 2014059, Warsaw, Poland) has a 16 MHz central frequency, a fractional bandwidth equal to 0.25 at −6 dB, and was used in the pulse-echo mode. The fabricated needle-type probe has a 3 mm external diameter with a 2 mm active piezoelectric element. The ultrasound technique used for the detection of the inclusions was based on the reflectometric method, which avails of the signals reflected by a stainless-steel plate supporting the excised tissues. The probe characteristics were selected for working in direct contact with the sample by means of controlled force indentations without any coupling means (ultrasonic couplant gel or water). This solution was necessary to avoid any contamination of the sample, to be further analyzed by pathologists. The ultrasonic coupling with the probe was assured by the natural wet surface of the sample. We have chosen the needle probe with a diameter small enough in order to have a good spatial resolution for the tissue mapping, but sufficiently large to avoid sample damage due to the applied pressure necessary for the tactile sensing.

The ultrasonic analysis consisted of the processing of the signal detected in each point of the indentation matrix as described in detail in Section 3.

### 2.2. Design and Manufacture of Agar-Phantoms with Agar-Spherical Inclusions

Preliminary tests were performed on an agar block-shaped phantom already used in the previous experimentation, which was realized to mimic both the mechanical and the acoustic properties of diseased human tissues [18]. This was done in order to validate the new ultrasonic analysis approach on phantoms before using the procedure on samples of liver tissue. The agar phantom had a soft surrounding matrix representing the human healthy tissue and harder agar inclusions embedded inside that represent tumor nodules. The phantom was nominally 60 mm wide, 100 mm long and 15 mm thick, while the buried spherical inclusions had diameters of 3, 6, 9 and 12 mm. The centers of the inclusions were positioned at half depth, i.e., about 7.5 mm from the surface, in the near field of the probe. This situation may complicate the intensity analysis for the amplitude variations of the propagating beam especially for the biggest inclusion, which is closest to the surface, but it is realistic for the application, and it was tested for first. 

### 2.3. Design and Manufacture of Liver Tissues Phantoms with Agar Inclusions with Non-Regular Geometrical Shape

We designed phantoms of liver tissues using the pork liver because it’s more similar to a human liver than other types of animals’ liver. The width and length of these phantoms varied between 30 to 40 mm with a thickness of about 15 mm. We mimicked the cancerous nodules by injecting a mixed solution of 8 g of agar in 100 mL of water in the excised tissues by means of a syringe provided with a conic hollow. Finally, we obtained the following inclusions with different shapes and dimension embedded in the tissue:

1) Inclusion type I: Rather spherical inclusions of small dimensions (typically 2–3 mm wide) obtained introducing a small amount of solution within the intact liver;
2) Inclusion type II: Extended inclusions which filled almost entirely the channel produced by the syringe tip inserted into the tissue. They were obtained going on injecting a solution in the tissue while extracting the syringe;
3) Inclusion Type III: Inclusions with the form of the open vessels (vein or artery) that we found in the dissection of the liver tissues and we utilized as a tube to be filled from the outside with the agar solution.

These agar inclusions rapidly solidify within the tissue as soon as we injected the solution. Many of them contain air bubbles like the agar spheres in the phantom matrix did, while their surfaces are rarely smooth and spherical. After dissection, the inclusions of type I were rather flat. In fact, the internal meshes existing within the liver tissues tend to impede the formation of smooth and perfectly spherical shapes. Also, the inclusions of type II and type III were rather flat. We observe that the air bubbles are only present in the agar inclusions but not in human tissues; their effect is to create additional attenuation in our experiments. However as shown later in the paper, the results showed that they did not impede to obtain a good detection performance of the inclusions. The external shapes of these inclusions have the smoothest external surfaces for the nature of the walls with which they were in contact. Some of the inclusions are shown in Section 4.2 reporting the experimental results after having cut the liver tissues into two parts to reveal the actual position and spread of the inclusions.

The experimental protocol for both types of phantoms (agar-based with spherical inclusions and liver tissue phantoms) consisted of the automatic scanning of the samples. For first, the sample was visually inspected to recognize the boundaries by means of a video camera [18] and to create the indentation matrix, namely the arrays of points to be indented and analyzed. Once the visual analysis was completed, it was possible to start the acquisition of the ultrasound signals. For each X–Y point of the indentation matrix, the phantom was indented along the Z-axis at a constant speed (0.5 mm/s) for a total number of *n* indented points using a spatial resolution of 2 mm. The indentation force was continuously recorded and, at the threshold level of 0.2 N, a trigger signal was generated for the ultrasonic pulse transmission and the successive signal recording upon the reception of the reflected pulse. The threshold value was experimentally determined to avoid damaging of the phantom or tissue.

## 3. The Data Analysis Approach for Detection and Localization of Inclusions Based on the Combination of Correlation Indexes Related to Shape and Amplitude of Reflected Signals

As mentioned in the previous sections, the ultrasound technique, used for the detection of the inclusions, is based on the pulse-echo method [13,14,15,16] processing ultrasonic signal reflected from a stainless-steel plate where phantoms are positioned. In the phantoms, the reflected signals passing through the inclusions had a lower amplitude than those reflected from the plate that had not passed through any inclusion. In the agar phantoms, the reflected signals from inclusions were very small, lower by about two orders with respect to the amplitude of the signal reflected from the plate. Hence, these small signals were not considered in our analysis, but only the intense signals reflected by the stainless-steel plate were considered having a higher Signal to Noise Ratio (SNR). The reflected signals resulted in delays according to the sample’s thickness, which could be slightly different in the different probe positions. Moreover, to have full control of the experimental parameters, the sample’s thickness is measured and recorded with high precision through the Z-axis of the robotic platform. In [18], the ultrasonic analysis consisted of the processing of the signal detected in each point of the indentation matrix by using a CIA parameter that we report in Equation (1):

(1) $$\mathrm{CIA}=1-\left(\frac{\min\left(\sqrt{\sum S_{ref}^{2}},\sqrt{\sum S_{i}^{2}}\right)}{\max\left(\sqrt{\sum S_{ref}^{2}},\sqrt{\sum S_{i}^{2}}\right)}\right)$$

In Equation (1), *S_i_* is the signal acquired in each scanned point (*i*-th point) and *S_ref_* is a reference signal. We assumed that a reference signal could be acquired in a position where neither inclusions nor other inhomogeneity has been detected. In the experimentation, we checked different points of this type. The CIA parameter value ranged between 0 and 1. It is zero for two signals with identical amplitudes, while it approaches 1 for very different amplitude signals. A CIA [1, *n*] color map was obtained for all scanned points *n*. The number of scanned points varies among samples in consideration of their dimensions and shape. The grid of points is obtained from the X–Y rectangular area selected as shown in detail in Section 4.2. The dimensions of the CIA color maps are relative to the X–Y scanned area. In our agar-phantom test samples, we found a great amplitude variation relative to the ultrasonic signal acquired outside the inclusions respect to that acquired over the inclusions. This variation is about 60%. Other experiments carried out on some types of liver tissues (i.e., from rabbit, chicken and ox) showed smaller amplitude variations of only 20% for the echo signals acquired outside and over inclusions.

In the CIA analysis reported in [18], we assumed that the signals from the US probe were only attenuated for the travel path from the agar sample surface to the steel plate interface and back. This simplified assumption did not take into account the diffraction effects that influence the US signals’ shape. 

**Figure 2:**
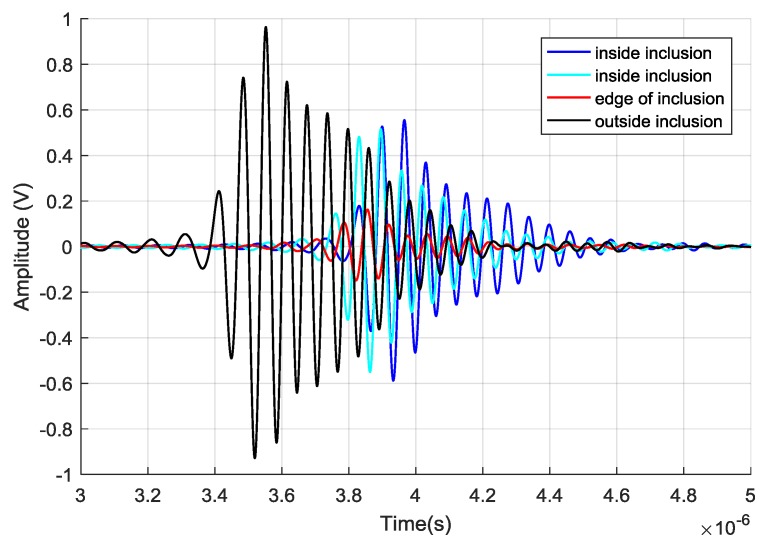
Raw data of the received signal as a function of time in different places of the phantom: outside (black line), inside (light blue and blue lines) the agar inclusion of 12 mm in diameter and at the edge of it (red line).

**Figure 3:**
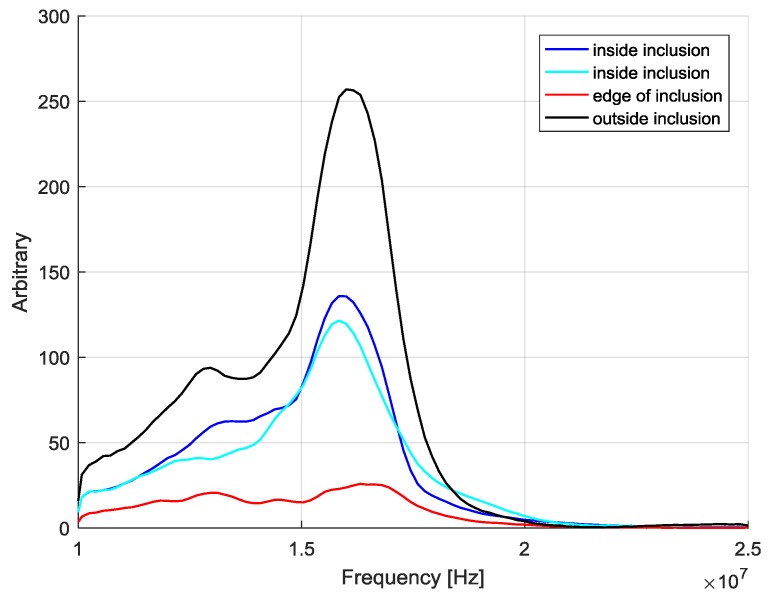
The frequency spectrum of the four signals shown in Figure 2.

In Figure 2, we report four of these reflected signals for phantom outside (black line), inside (light blue and blue lines) the agar inclusion of 12 mm in diameter and at the edge (red line). Those inside show higher attenuation than outside and a slight modification of the high-frequency content of the signal spectrum. On the other hand, the signal in correspondence of the edge shows a drastic reduction in amplitude, especially for the cycles at the highest frequencies and consequently a more pronounced shape distortion. The Fast Fourier Transform (FFT) of the four signals reported in Figure 3 clearly underlines this behavior.

Specular reflections did not produce shape envelope distortion, and we did not expect a non-linear behavior from agar, but only an attenuation that varies with frequency according to the variation of the absorption coefficient [24] for the same depth. On the contrary, in the presence of diffraction [15,25,26], objects with a dimension larger than the wavelength can bend the incoming wave, forming a beam pattern with double peaks. We can also observe that the low-frequency components of the spectrum of the transmitted signal could cross the target more easily according to the laws of diffraction and then modify the shape and amplitude of the echo signal. Hence, the signal shape deserved to be considered to improve the analysis capabilities in finding inclusions. The shape modification of the reflected signal was introduced by considering a CIS parameter defined in Equation (2) [27]:

(2) $$\mathrm{CIS}=1-\frac{\sqrt{{\left(\sum S_{ref}S_{i}\right)}^{2}}}{\sqrt{\sum S_{Bref}{}^{2}\sum S_{i}{}^{2}}}$$

**Figure 4:**
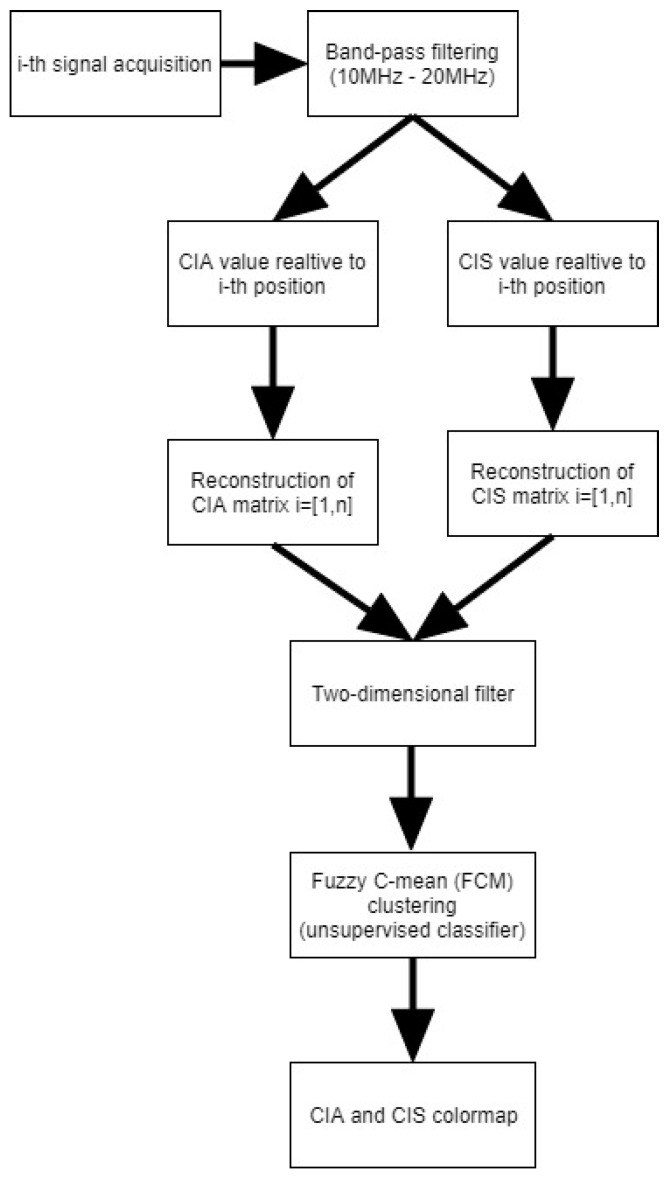
Flow-chart relative to Correlation Index Amplitude (CIA) and Correlation Index Shape (CIS) processing for the ultrasonic signal.

According to the introduction of the CIS parameter, in Figure 4, we report the flow chart of the new signal processing relative to the use of both CIS and CIA.

For the automatic detection of inclusions, we applied also the AND-OR logical operators to the CIA and CIS results.

## 4. Experimental Results

The CIA and CIS results of one automatic scan performed for the acquisition of the ultrasonic signals from the agar phantom are presented in Section 4.1. The following Section 4.2 reports the same CIA and CIS processing for signals detected in pork liver samples with agar inclusions. In all the experiments, we carried out the indentation matrix in X–Y directions at steps of 2 mm, the same dimension of the active piezoelectric element. Considering only these two geometrical aspects, we would expect a maximum error in the determination of the inclusions’ positions equal to about this value.

### 4.1. Results on Agar Phantoms

**Figure 5:**
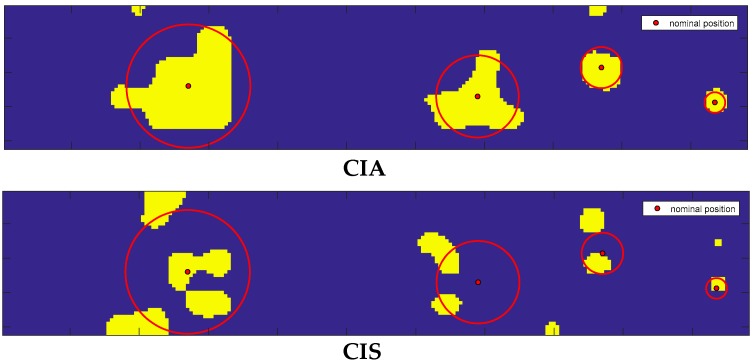
Map of inclusions on an area of 72 mm × 14 mm corresponding to *n* = 252 scanned points. (Top) CIA map and (Bottom) CIS map. Dimensions of the four inclusions are (from left to right): 12, 9, 6 and 3 mm. Red dots represent the nominal position of inclusions and red circles their dimensions. Yellow color indicates the areas over which the unsupervised classifier algorithm found inclusions while the blue color indicates those where the algorithm did not find inclusions.

In Figure 5, the color map for CIA and CIS are reported oversampling the area of the indentation matrix with a step of 0.33 mm to improve both images and classification operations.

The red “circles” relative to the inclusions, with their center also reported in red, were determined by superimposing the color map on the indentation matrix. Based on the CIA result we could expect a yellow color for the pixels inside inclusions. Furthermore, based on the US signal’s different attenuation, which is greater over bigger inclusions, we could expect a CIA map with larger yellow regions inside the red circles for bigger inclusions. On the contrary, there are more regions inside the two larger red circles that are falsely classified as background due to any of the following causes: diffraction effects [25,26], the presence of defects in the inclusions (voids or air bubbles), a possible tilt angle of the probe needle and movements of the inclusions during the probe indentation phase. An overall error can be estimated by means of the knowledge of the centers of agar inclusions, since they have specified shape and position in the matrix. The error estimated in this way would be specific only of these particular points, notwithstanding they may have a certain significance in the nodules’ individuation also. According to previous research [28], we individuated these centers’ positions from the CIA data and compared them with their nominal positions. With reference to Figure 5 (top), we found that these differences were smaller than the scanning spatial resolution of 2 mm. 

CIS analysis was introduced to put in evidence the signal shape distortions, also those deriving from the boundary regions in the near field. These regions produced a yellow area in the CIS map close to the center of the biggest inclusion, whose upper central part was closer to the probe with respect to all the parts of the other inclusions.

**Figure 6:**
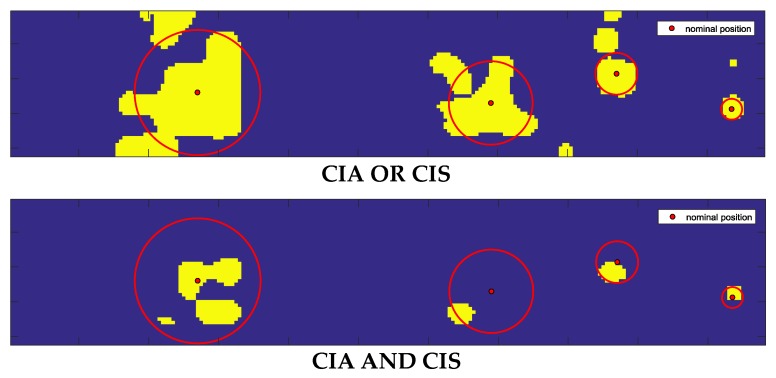
OR-logic map (Top) and AND-logic map (Bottom) from CIA and CIS data shown in Figure 5.

The results obtained from the application of the AND-OR logical operators are shown in Figure 6.

One advantage of considering the CIS parameter in combination with CIA derived from the fact that these contained different information. 

Figure 6 (Top) shows the map obtained with the application of the OR operator to the CIA and CIS values reported in Figure 5. It can be observed that the CIS parameter contributes to decreasing the number of false negatives (FN) represented by the blue pixels inside the inclusions, with a small increment of false positives (FP) represented by the yellow pixels outside the inclusions. The smallest inclusion on the right is perfectly identified. The identification of this inclusion confirms the capabilities of ultrasound to detect an object of small dimensions.

### 4.2. Results on Liver Tissue Phantoms

Human tissues have different textures with respect to the realized agar phantom. The US detects these differences in terms of complex changes in amplitude and shape of the reflected back signals due to diffraction and scattering phenomena. The background effects were investigated with a study for both CIS and CIA on tissue samples before the introduction of the agar inclusions, which also can contain internal structures with significant echogenicity. A preliminary analysis was performed with the Esaote MyLab™X7 ecographic equipment using a linear array ultrasound transducer SL3116 having a central frequency (15 MHz) close to the needle probe integrated into the robotic platform. The analysis of the US image shows the complexity of the internal structures of the liver in order to justify the large variation of the CIA and CIS parameters we found in our preliminary experiments.

**Figure 7:**
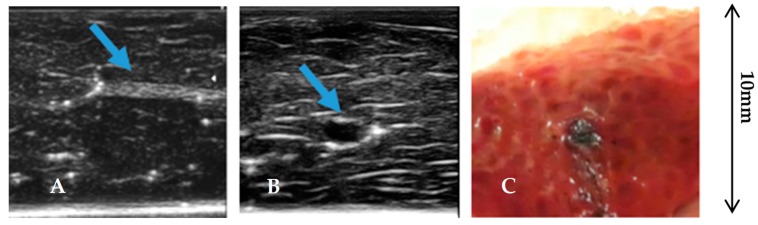
B-scan image of the internal structure of the liver sample (**A**) using a linear array ultrasound transducer SL3116. (**B**) B-scan image of the liver acquired over a 2 mm agar inclusion using the same linear transducer; (**C**) picture of the liver slice obtained dividing the tissue into two pieces in correspondence of the inclusion.

We report an image of one of them in Figure 7A, and the image of a small inclusion of agar in Figure 7B. This inclusion is about 2 mm large; its dimension represents in practice the detectability limit by palpation for a skilled physician. In Figure 7C, we show an image of the liver slice obtained dividing the tissue into two pieces in correspondence of the small agar inclusion. This practice was utilized to analyze all the types of inclusions we made for evaluating their edges.

After this preliminary analysis, which was necessary to highlight the complexity of the internal structure of pork livers, we carried out experiments on three liver tissue samples using the data analysis approach reported in Section 3.

**Figure 8:**
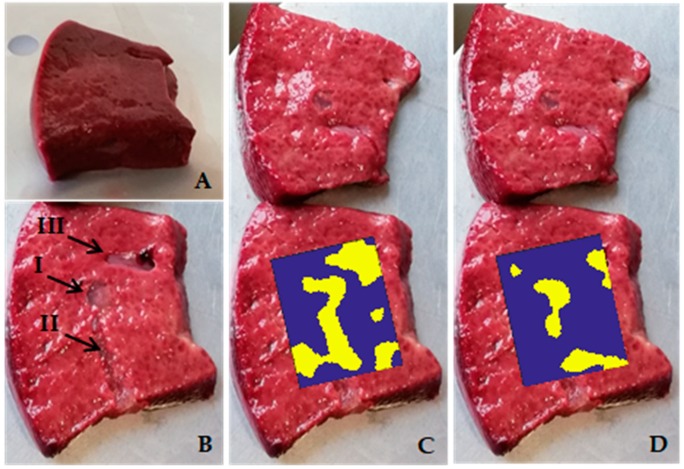
Analysis of the liver tissue sample #1. (**A**) picture of the liver sample with the agar inclusions inside. (**B**) One of the two slices of the sample #1 with marked inclusions of type I, type II and type III. (**C**) CIA and (**D**) CIS maps superimposed on the scanned area.

Sample #1 liver tissue is reported in Figure 8A. In Figure 8B, the inclusions of type I, II and III are clearly visible after having cut the tissue into two pieces. The CIA map is reported in Figure 8C and the CIS map is reported in Figure 8D.

**Figure 9:**
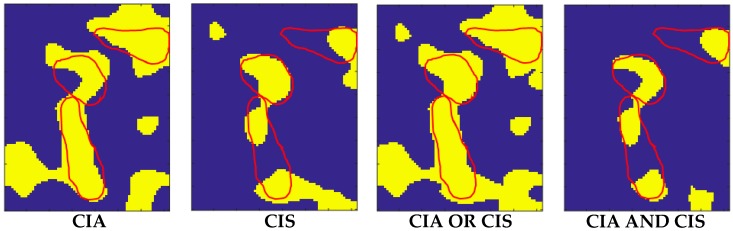
From left to right: CIA, CIS, OR-logic and AND-logic maps for liver sample #1. The dimension of the scanned area was 24 × 30 mm^2^. The total number of scanned points in the investigated area was *n* = 180.

For a more detailed analysis of results, the CIA and CIS maps and the AND-OR logic maps of sample #1 are reported in Figure 9 with the evaluated margins of the inclusions reported in red. The contours of the inclusions in the liver were determined by superimposing the color map on the picture of one of the tissue slices into which the sample was cut (Figure 8B). The pictures of the CIA and CIS color maps are shown in Figure 8C,D, respectively.

**Figure 10:**
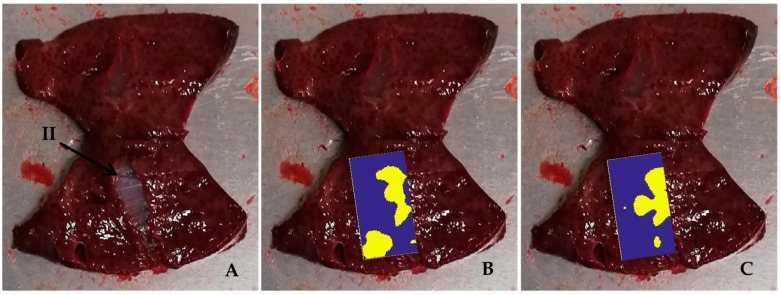
Analysis of the liver tissue sample #2. (**A**) Sample divided into two pieces with marked inclusion of type II; (**B**) CIA and (**C**) CIS maps overlapped to the scanned area over the inclusion.

Results obtained on a second liver tissue examined (sample #2) are reported in Figure 10. Figure 10A represents sample #2 divided into two pieces. Figure 10B,C show the CIA and CIS maps, respectively, positioned on one of the two slices.

**Figure 11:**
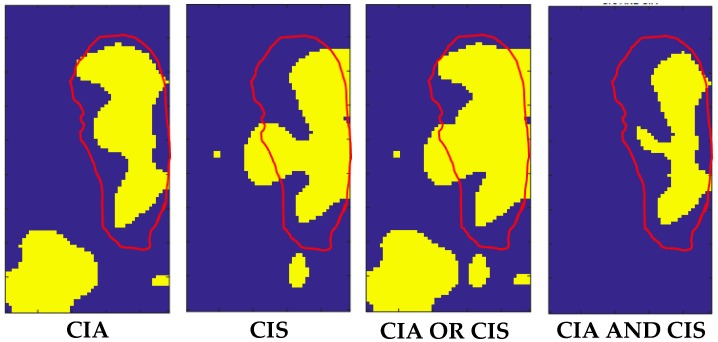
From left to right: CIA, CIS, OR-logic and AND-logic maps for liver sample #2. The scanned area was 16 × 30 mm^2^. The total number of the scanned points was *n* = 120.

The analysis of the CIA map, CIS map and AND-OR logic maps of sample #2 are reported in Figure 11.

In the following, we used the terms True Positive (TP), True Negative (TN), False Positive (FN) and False Negative (FN) for the classification of the sub-pixels in all the maps that used the evaluated edges (red lines in the figures) of the inclusions. 

The results in Figure 9 show that we could detect all the inclusions from both CIA and CIS maps, but in the CIS map, the FP region appears less pronounced than in the CIA map. This is confirmed also by the AND-logic map while in the OR-logic map, the FN region appears less wide than the FN region in the CIA map, with a small increment of the FP area. 

Elaboration of sample #2 confirmed that the OR-logic application increased the TP area and decreased the FN area. Results on both samples demonstrated that CIS reduced the FP area with respect to use only of CIA. The results presented in both Figure 9 and Figure 11 indicate that the AND-OR logic can be considered complementary in the determination of the shape of the inclusions. 

The structures of the liver can contribute to the FP outside the edges of inclusions, while discontinuities in the agar inclusions can contribute to the increase of the FN inside inclusions. Notwithstanding these drawbacks, we found a reduction of the FN area thanks to the introduction of CIS in combination with CIA. This improvement can be observed from Figure 9 and Figure 11, where the areas individuated by the AND logic are in good agreement with the core of the inclusions, while the areas deriving from the OR logic have a significance for including most of their edges.

### 4.3. Confusion Matrix Analyses

The confusion matrix reported in Table 1 was obtained using the results of the CIA, CIS and AND-OR logics performed on three liver tissues, for a total number of 13,608 classified points (378 scanned points). The total number of True Positive (TP) was 2545 and the total number of True Negative (TN) was 11,063. Each point corresponded to an area of 0.33 × 0.33 mm^2^.

Observing the confusion matrix reported in Table 1, the CIA analysis presents valuable classification results in detecting the inclusions with 77.64% of TP and 87.37% of TN. Moreover, it also shows a not negligible percentage of FP and FN, which resulted to be 12.63% and 22.36%, respectively. On the contrary, the CIS classification presented a reduced TP percentage (64.05%), and a higher amount of TN (97.97%) with respect to those found from the CIA analysis. The very small percentage of FP (2.03%) was the most interesting result deriving from the CIS analysis. 

To improve the performance, the classified datasets were logically merged using the OR-AND logics. As expected from the results reported in Figure 9 and Figure 11, the OR logic gave evidence of a higher rate of inclusions recognition (i.e., 84.57% of TP and 84.45% of TN), while maintaining low the error rates (i.e., 11.43% for FN and 15.55% for FP). Such a result is a direct consequence of the implementation of this logic, since we considered all the points classified as inclusions in both the CIA and CIS datasets. This fact entails also a better localization of the buried inclusions. On the other hand, the AND logic localized all the inclusions with an increased percentage of TN up to 99.10% and a reduced percentage of the FP down to 0.90%, with low TP and FN rates of 53.05% and 46.95%, respectively. 

## 5. Conclusions

In this work, we presented a strategy for the detection and localization of nodules in liver tissues by a 16 MHz needle ultrasonic probe mounted on a robotic platform. The cancer nodules were mimicked using an agar mixture. Several tests were performed in a laboratory environment using three liver tissue samples. Preliminarily, the method was verified on one of the agar phantoms. The inclusions were designed using agar injected into liver tissues. They had non-regular geometrical shapes mimicking tumor alterations in healthy tissue. The inclusion detection strategy was based on the intensity and shape modification of the ultrasonic signals, using two parameters (CIA and CIS). We considered the results obtained from the application of the AND-OR logical operators to these two parameters to improve the recognition possibilities of inclusions. The overall evaluation of the performance of the new processing strategy, tested on liver tissue samples, were obtained by using the confusion matrices already adopted in [18] for agar phantoms. The main result deriving from the liver tissue analysis was the demonstration of the ability of the ultrasonic method to automatically individuate the inclusions by mapping the scanned area, particularly when combining CIA and CIS parameters via OR logic. This method can also be a viable solution to facilitate a pathologist in decision-making by accessing this output remotely, by appropriate software interface. In particular, the OR-logic analysis presented valuable classification results in detecting the inclusions, achieving an 88.57% of TP and 84.45% of TN. Moreover, it showed a low percentage of FP and FN, i.e., 15.55% and 11.43%, respectively. This type of analysis could deliver to the pathologist the areas of interest over which perform the analysis. On the other hand, the AND logic analysis was valuable for the high number of TN (i.e., 99.10%), and consequently for the capacity of individuating a high-risk region within the area of interest. However, since it provided only a partial shape recognition of agar inclusions mimicking tumors, it still requires the integration with the OR logic results, or with the tactile analysis, in an automatic measurement processing to be implemented on a microprocessor onboard the robotic platform control unit. Since the numerical complexity of the proposed signal and image processing was moderate, the state-of-the-art digital signal processors (DSP) can provide the results in an order of seconds. Such a feature is important for the final application and can envisage a new pre-clinical research step on human dissected tissues. 

## Figures and Tables

**Table 1:** Confusion matrix with classification based on CIA, CIS and AND-OR logic analysis.

**CIA**	
**9666**	**1397**
87.37%	12.63%
**TN**	**FP**
**569**	**1976**
22.36%	77.64%
**FN**	**TP**
**CIS**	
**10838**	**225**
97.97%	2.03%
**TN**	**FP**
**915**	**1630**
35.95%	64.05%
**FN**	**TP**
***CIA* AND *CIS***	
**10963**	**100**
99.10%	0.90%
**TN**	**FP**
**1195**	**1350**
46.95%	53.05%
**FN**	**TP**
***CIA* OR *CIS***	
**9343**	**1720**
84.45%	15.55%
**TN**	**FP**
**291**	**2254**
11.43%	88.57%
**FN**	**TP**
